# Design and Construction of a Micro-Tribotester for Precise In-Situ Wear Measurements

**DOI:** 10.3390/mi8040103

**Published:** 2017-03-28

**Authors:** Oleksiy V. Penkov, Mahdi Khadem, Andy Nieto, Tae-Hyeong Kim, Dae-Eun Kim

**Affiliations:** 1 Center for Nano-Wear, Yonsei University, Seoul 03722, Korea; penkov@yonsei.ac.kr (O.V.P.); mehdi.skyline@yonsei.ac.kr (M.K.); andynieto15@hotmail.com (A.N.); kth1990@yonsei.ac.kr (T.-H.K.); 2 Department of Mechanical Engineering, Yonsei University, Seoul 03722, Korea; 3 Vehicle Technology Directorate, Army Research Laboratory, Aberdeen Proving Ground, MD 21005, USA

**Keywords:** in-situ examination, data acquisition, signal processing, friction, wear

## Abstract

Extensive research efforts have been devoted to understand the complex mechanisms of wear with the aim to minimize wear in sliding systems. Improvements in the instruments used for the characterization of the wear phenomenon are required to enhance the effectiveness of research method. In this paper, we report the design of an experimental platform that enables in-situ observation of the surface topography evolution during the evaluation of the tribological behavior of surfaces in dry and lubricated conditions. Use of state-of-the-art components for surface topography measurement, planar positioning, and force sensing allowed for the improvement of sensitivity and resolution compared with the previously reported systems. The effectiveness of the tribotester was demonstrated through friction and wear tests performed using a stainless-steel ball and a silicon wafer coated with SiO_2_. It was found that transition of the wear mechanism from adhesive to abrasive wear took place when a significant amount of wear debris was formed as evidenced by the in-situ observation of removal of the coating and exposure of the Si substrate. The in-situ observation of wear phenomena enabled a robust and in-depth elucidation of wear mechanisms.

## 1. Introduction

Wear is a consequence of the prolonged frictional interaction between two contacting surfaces in relative motion. Wear is often a critical issue in various industries including automotive, machinery, aerospace, biomedical, and micro- and nano-electromechanical systems (MEMS and NEMS) [1,2,3,4]. A better understanding of the fundamental processes of friction and wear requires the characterization of engineered surfaces not only after completion of the wear test but also during the wear test to understand the transitional wear behavior that led to the final state. A pin-on-disk or reciprocating tribotester and a topographical microscope are common assets for tribological experiments. In a typical experiment, the specimens are subjected to frictional loads, and then the surface topography is examined using a topographical microscope. Depending on the scale of the experiment, the examination of the wear track can be done using 3D profilometry [5], scanning electron microscopy (SEM) [6], or in the case of micro- or nanoscale friction, via atomic-force microscopy (AFM) [7,8,9,10]. It should be noted that assessment of wear by optical microscopy or SEM cannot provide sufficient quantitative information about surface topography, and hence utilization of 2D/3D profilometry techniques is necessary for quantitative characterization of wear. Though normal and lateral forces being commonly recorded during a tribological test, the surface topography and wear are typically assessed after completion of the wear test using conventional instrumentation. Therefore, information about the dynamics of the wear process cannot be readily obtained. This issue can be partially resolved by using a transparent counter surface that enables direct observation of the contact pair [10,11]. Such a technique was also utilized by Kim et al. [12] to investigate the wear mechanisms of graphene. However, this approach has significant practical limitations because most of the materials that are used in tribological applications are not transparent.

In-situ characterization techniques can be used for the identification and detailed examination of various phenomena taking place in mechanical, chemical, and electrical engineering systems [13,14,15,16,17]. This technique can also be utilized for the investigation of tribological phenomena. The concept of an in-situ tribotester was proposed by Wahl and Sawyer [18]. A reciprocating tribotester was mounted onto a scanning white-light interferometer to observe the wear surface as sliding progressed. Use of such an apparatus allowed for the correlation the variation of the coefficient of friction with the wear behavior during the sliding test. The formation of wear debris and deep scratches could be correlated to an increase in the coefficient of friction. Korres and Dienwiebel [19] combined a pin-on-disk tribotester with a three-dimensional holography microscope that enabled direct observation of the surface modification during the tribological experiment. Although the proposed method was successfully used for the characterization of wear of lubricated metallic surfaces, it had significant disadvantages. Use of the holography microscope requires utilization of the radionuclide technique and thereby limits the applicability of this method due to safety concerns. Also, it is suitable for characterization of wear under lubricated conditions only. High precision 3D laser microscopy overcomes these shortcomings of holographic microscopy and has been successfully used for the detailed quantification of wear with nanometric resolution as demonstrated by Khadem et al. [20] and Park et al. [21].

In this work, a tribotester enabling in-situ monitoring and evaluation of wear under dry and lubricated conditions was developed. The main goal of the experimental setup was to provide continuous assessment of the wear phenomenon during the sliding test to obtain the information about the wear mechanism in progression [18]. This could be achieved by monitoring the state of a given location in the wear track through the viewport of the 3D laser microscope. Such configuration allowed to evaluate the wear process in detail during each sliding cycle. Thus, it was possible to investigate the wear transition processes with respect to the sliding distance. Use of state-of-the-art components for surface topography measurement, planar positioning and force sensing allowed to improve the sensitivity and resolution compared with the previously reported systems. The following sections describe the details of the instrument design, construction, and usage.

## 2. Experimental Details

### 2.1. Principle, Design, and Function

A high precision 3D Laser Microscope (LM, VK-X210, Keyence, Tokyo, Japan) was used for the measurement of the surface topography with a planar resolution less than 0.01 µm and a height resolution of 0.005 µm. The microscope was equipped with a motorized *XY*-planar actuator with a scanning range of 100 mm × 100 mm and a resolution of 5 μm. The tribotester was assembled as an autonomous unit and then attached to the motorized *XY* planar actuator of the microscope. The actuators of the microscope were used for precise positioning of the tribotester system under the lens of the laser microscope. The load capacity of the microscope actuator was 5 kg, which was sufficiently high for supporting the tribotester weight without compromising the positioning accuracy or disrupting the performance of the actuators. The positioning of the tribotester directly under the objective lens of the microscope enabled direct observation of the contact point between the specimen and the pin which is used as a counter surface.

**Figure 1:**
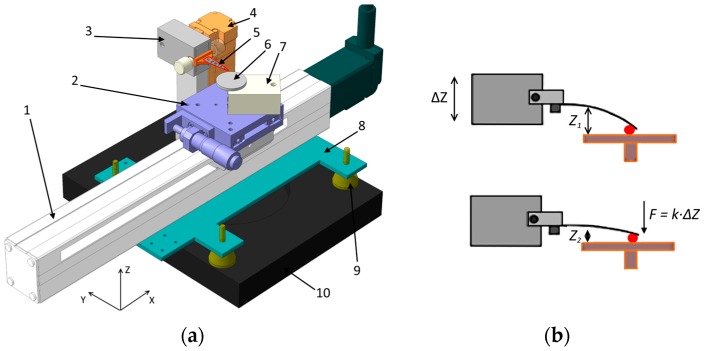
Schematic of the tribotester: (**a**) Schematic of the design: (1) reciprocating *X*-axis actuator; (2) manual *Y*-axis stage; (3) frictional force sensor; (4) *Z*-axis actuator; (5) counter surface suspension (cantilever); (6) specimen holder; (7) normal force sensor; (8) chassis; (9) elastic shock absorbers; (10) *XY*-motorized actuator of the microscope; (**b**) Schematic of the generation and control of the normal force.

The design of the tribotester system is shown in Figure 1a. Tests were conducted by reciprocating the test specimen in contact with a tip under a specified normal force. The reciprocating motion along the *X*-axis during the friction test was generated by a high-precision linear actuator (EasyLimo, Oriental Motor, Tokyo, Japan). This actuator has a stroke of 200 mm and a maximum reciprocating speed of 300 mm/s. The position was controlled with a resolution of 20 μm. The actuator was attached to an aluminum chassis (8). This chassis was mounted on the *XY*-planar actuator of the microscope (10) through four polyurethane shock-absorbers (9). The length of the tribotester *X*-axis actuator was ~65 mm longer than the width of the *XY* actuators. Long *X*-axis allowed the specimen holder to be positioned away from the microscope during manipulation of the specimen holder to preserve the optics of the microscope. The manual stage (2) (PADRL-20, Oriental Motor) was attached to the top of the *X*-axis actuator (1). This stage was used for adjusting the position of the contact point along the *Y*-axis of the test specimen. The *Y*-axis manual stage has a stroke of 30 mm and a resolution of 10 μm.

Two independent sensors were used for the measurement of normal and frictional forces. In this case, two same force sensors were used. High-precision commercial load cells (strain gages, GS0-10, Transducers Techniques, Temecula, CA, USA) were utilized for this purpose. The sensing range of the load cell was 0.1–300 mN. The normal force sensor (7) was attached to the top of the *Y*-axis actuator. The specimen holder (6) had a threaded cylindrical pin that was inserted into corresponding holes in the normal force sensor. The test specimens were attached to the specimen holder using a two-sided adhesive tape, which was sufficiently strong to ensure that the specimens were rigidly attached and remained static during the sliding tests. The specimen holder could be easily detached from the normal force sensor to provide safe manipulation of the specimens and to avoid overloading the sensor. The frictional force sensor (3) was attached to the *Z*-axis actuator (4). The actuator had a 28-mm stroke and a resolution of 2 μm. The *X*- and *Z*-axis actuators were controlled with a programmable 2-channel controller (PMC-2HSP, Autonics, Seoul, Korea).

The normal force was generated by the elastic deformation of a steel cantilever suspension (5) attached to the frictional force sensor using a custom-built holder. The holder was attached to the frictional force sensor by two knobs that enabled quick replacement of the suspensions between experiments. In tribological experiments, a new tip is typically used for every experiment to ensure geometric integrity as the tip may also become worn during the experiment [6]. Hence, the ability to quickly replace the suspensions significantly increased the usability of the tribotester.

The suspension had a triangular shape in the plane, and the tip (counter surface) was attached to the end of the suspension with epoxy adhesive. Figure 1b illustrates the generation of the normal force schematically. By moving the frictional force sensor (3) downward using a linear actuator (4), the suspension was made to contact the specimen surface, and the normal force was generated due to the elastic deformation of the suspension. The amount of force was dependent on the spring constant (*k*) of the suspension and vertical displacement (Δ*Z*) of the tip holder. The maximum force was limited by both the sensing range of the load cell and the elastic deformation of the suspension. The normal and frictional force sensors were connected to a commercial multichannel analog to digital converter (ADC NI DAQ 9420, National Instruments, Austin, TX, USA) unit using the full-bridge scheme. The ADC resolution was 24 bits, and the sampling rate was 100 kHz.

### 2.2. Software and Control of the Tribotester

**Figure 2:**
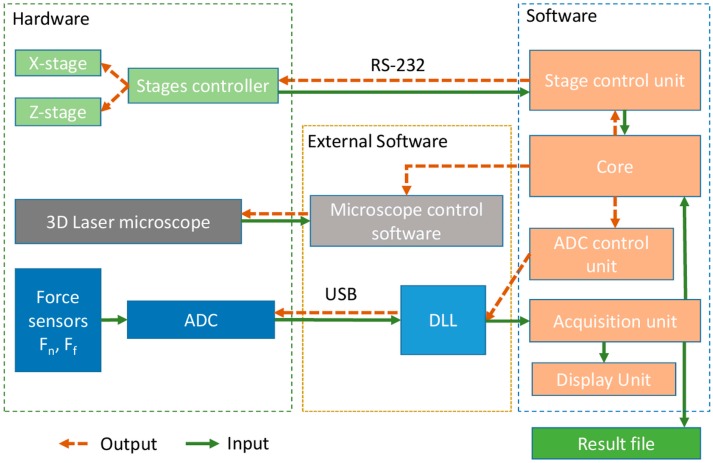
Software and control diagram of in-situ tribotester.

A single workstation controlled the operation of the tribotester. The microscope was controlled by the software supplied by the manufacturer. The friction testing module was controlled by customized software developed by the authors. The control and software diagram for the tribotester is shown in Figure 2. The core controlled the other components based on signals received from the other units and the operating system. The actuator control unit received commands from the core and transmitted them to the programmable actuator controller. The function of the actuator controller was to send responses containing the information about its status and the positions of the actuators.

The acquisition unit read the buffer and wrote the raw data to the resulting output file. Also, data was processed and sent to the display unit. The display unit roughly calculated the coefficient of friction values based on the measured values of the normal and frictional forces and displayed them on a graph in real time. The communication with the microscope software could be performed in automatic or manual mode. Automatic mode was based on the messages sent to the main window of the microscope control application. This mode enabled the measurement of the wear track topography in a fixed position and saved the results into a file. In the manual mode, the core paused the reciprocating motion and displayed a message for the operator to perform the measurement of the specimen wear track topography through manual operation. After performing the measurements, the operator may resume the reciprocating motion to continue the test.

### 2.3. Experiment Flowchart

**Figure 3:**
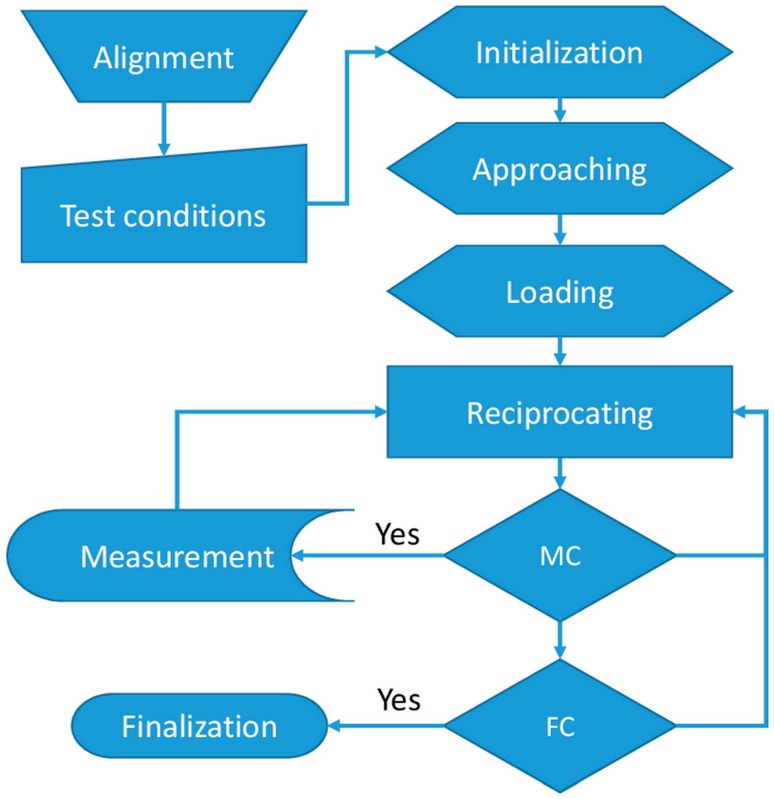
Experimental flow chart.

The typical experimental flow chart is shown in Figure 3. The preparation steps included attachment of the specimen to the substrate holder, mounting of the tip holder onto the frictional force sensor, and alignment of the tip position. The experimental conditions were fully adjustable and included stroke length, linear speed, the number of cycles, and wear track observation intervals. The experiment started by approaching the tip to the specimen. During this step, the *Z*-actuator was moved downward, and the force indicated by the normal force sensor was monitored continuously during the movement. The movement was stopped when a contact force between the tip and the specimen was detected. After the tip approach, the loading step was performed. The *Z*-actuator was moved downward until the target normal force was applied on the specimen surface by the tip.

The accuracy of the normal load applied to the specimen was dependent on parameters such as the approach speed and the sampling rate. To minimize the preparation time and to maintain high accuracy, the loading process was split into three steps. During the first step, the *Z*-actuator was moved with a relatively high speed of 1 mm/s until the normal force reached 75% of the target load. Then the speed was reduced to 0.1 mm/s until the load reached 95% of the target load. In the last step, the speed was reduced to 0.02 mm/s until the target load was reached. This three-step procedure enabled the precision of the normal force applied to the specimen to be below 0.5%.

After applying the normal force, the *X*-axis actuator was triggered to produce the reciprocating motion. After a specified number of reciprocating cycles, the reciprocating motion was paused to allow the investigation of the wear track surface topography. During the examination of the wear track by the microscope, it was important to assure that the tip or the counter surface maintained contact with the specimen. This allowed for the continuation of the test from the same position after the measurement of the wear track surface topography. These steps are repeated until the target total number of cycles would be reached.

### 2.4. Signal Processing

**Figure 4:**
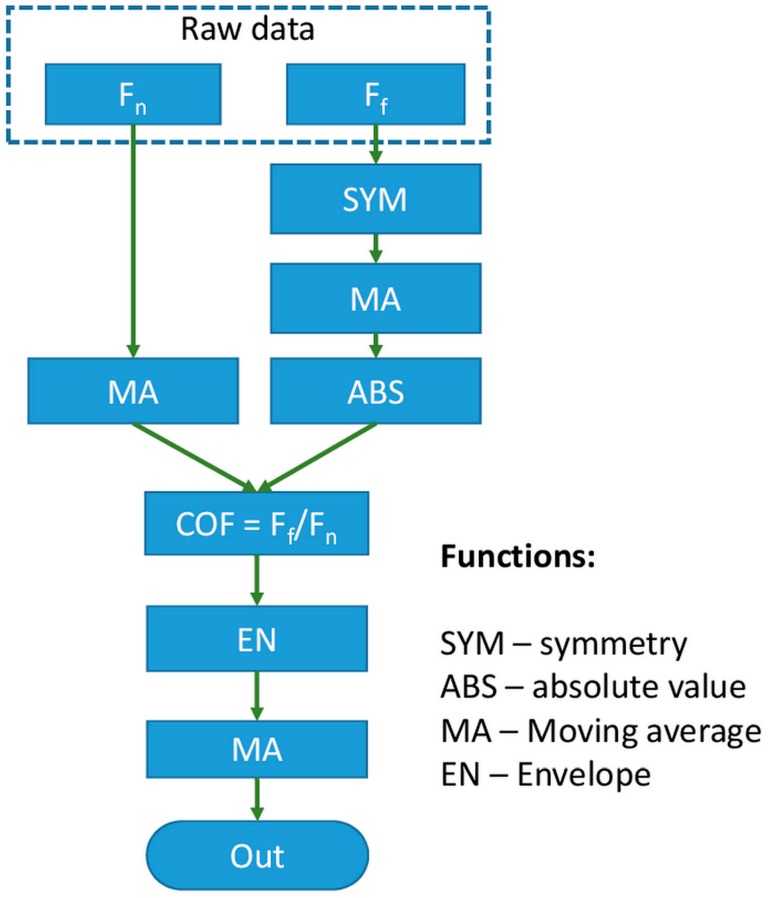
Diagram of the post-processing of the normal and frictional force raw data.

**Figure 5:**
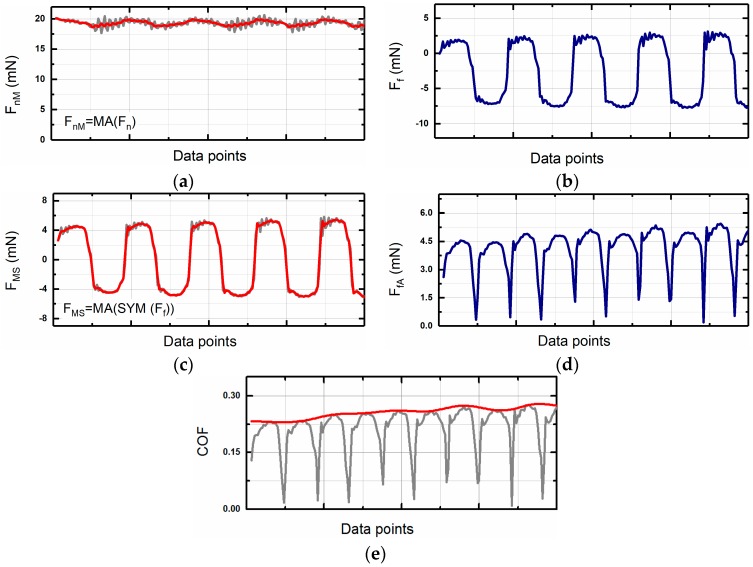
An example of the data before and after processing: (**a**) Raw and smoothed normal force signals; (**b**) raw frictional force signal; (**c**) symmetrical and smoothed frictional force; (**d**) absolute value of the frictional force; (**e**) coefficient of friction (COF) and envelope of COF.

**Figure 6:**
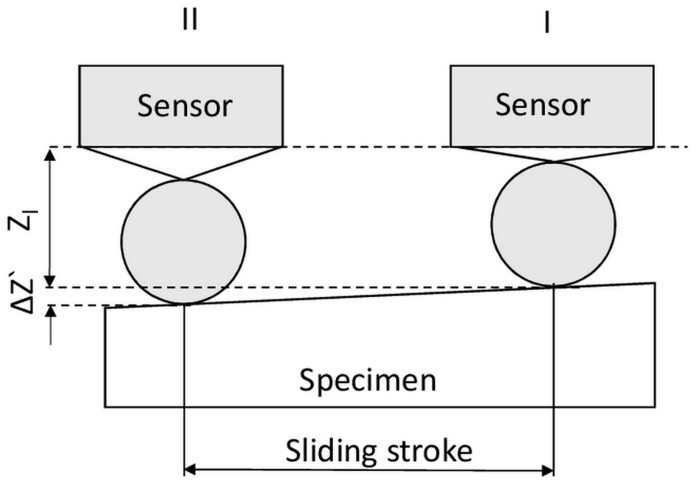
Schematic of the nature of normal force oscillations. Positions I and II show extremal positions of the tip on the specimen.

After the experiment, the raw data was post-processed by separate software to get the final values of the coefficient of friction. Figure 4 provides an example of the post-processing sequence for the normal and frictional force raw data. The exact sequence of the data processing could be varied depending on the purpose of the experiment. In this case, the purpose was to assess the variation in the coefficient of friction as a function of the sliding distance. Thus, the peak values of the coefficient of friction rather than its variation during a single sliding cycle were analyzed. Initially, the data for normal and frictional forces were processed separately. The normal force was processed by using the moving average (MA) function (Figure 5a). Large-period oscillations on the graph could be caused by the uneven flatness of the specimen or by misalignment of the specimen attached to the holder. Figure 6 presents a schematic of these phenomena when the specimen is not perfectly flat. Initially, after application of the normal force at the initial position I, the distance between the normal force sensor and the specimen is *Z_l_*. The initial position of the sensor after the tip approach is *Z*_0_, and hence the normal force in the position I can be expressed as

*F*_*n,*I_*= k∙(Z_l_ − Z*_0_*)* (1)

where *k* is the spring constant of the cantilever suspension. When the tip slides to position II, the absolute *Z*-position of the tip changes due to the inclination of the surface resulting in a vertical displacement of Δ*Z*’. Finally, the normal force at position II can be calculated by the following equation:

*F*_*n,*II_*= k∙(Z_l_ +* Δ*Z’ − Z*_0_*)* (2)

Therefore, when the tip slides from the position I to the position II, the normal force would be linearly reduced by Δ*F* which is expressed as:

*ΔF = k∙*Δ*Z’* (3)

As the pin returns from position II to position I, the force would be increased back to *F_n,_*_I_. Hence, oscillation of the normal force with the amplitude of Δ*F* is expected to occur for every sliding cycle as can be observed in Figure 5a. This effect could be minimized by the precise alignment of the specimen and the cantilever before the experiment.

The processing of the frictional force data required more steps than the normal force. Due to the nature of the reciprocating sliding, the frictional force changes its sign from positive to negative (Figure 5b). Changing of sign occurred due to the change in the direction of motion during the forward and backward strokes in a single reciprocating sliding cycle. Also, as can be seen from Figure 5b, the signal was not symmetrical on zero. Unsymmetric was attributed to the initial misalignment or bending of the tip holder. This non-symmetry could be easily compensated during the post-processing using the symmetry function (SYM). To compensate for the misalignment, the SYM function shifts the frictional force data by a certain offset value of Δ*F*. To calculate Δ*F*, the average value of all the data is calculated. The offset value is the deviation of the average value from zero which can be expressed as

(4) $$\Delta F=-\frac{\sum_{i=0}^{N}F_{i}}{N}$$

where *N* is the number of sampled data. After calculating the offset value, all the data was shifted by the adding the offset value to the initial values. The result of processing the original data by the SYM function and smoothing by MA (Figure 5c). Finally, the data obtained in the previous step were converted to absolute values by using the absolute value function (ABS) (Figure 5d).

After processing the normal and frictional force values, the coefficient of friction could be calculated. An example of the calculated values for the coefficient of friction is shown in Figure 5e (gray line). The oscillations on the coefficient of friction were attributed to the transient process caused by the reversal of the sliding direction at two ends of the reciprocating stroke. Thus, the maximum values of the coefficients of friction for every sliding cycle were considered as significant, and the crevasses in the data were ignored. The data was treated using the envelope function (EN) to exclude the effect of the crevasses [22]. However, another function such as the average function could be employed instead of the envelope function at this step depending on the experiment conditions and goal. The red line shows the resulting coefficient of friction in Figure 5e. To convert from the number of data points to the number of sliding cycles, the *X*-axis of this graph should be normalized based on the number of data points per cycle. The example given in Figure 5 corresponds to 50 data points/cycle.

### 2.5. Experiment Reliability and Limitations

Reliability of the experimental results was assured by using high-resolution components as well as statistical analysis of friction and wear data. Precision mechanical and electrical components with high resolution and repeatability were used to build the system. Furthermore, the uncertainty in the tribological data was minimized by statistically processing the data obtained from repeated experiments. For this purpose, the sliding tests were repeated at least three times using a new pin for each test on the fresh surface. The wear area was measured in at least three different locations along the wear track, and at least 100 line scans were averaged during each measurement to quantify the wear amount [23].

The lower working range of the instrument was limited not only by the resolution of the sensors, stages, and the microscope, but also by the degree of total wear. In other words, the wear track had to be deep enough to obtain a reliable data regarding the wear rate of the test specimens. This test condition was achieved by selecting the proper load range and number of cycles through preliminary tests.

Another important point to consider was the wear of the specimen or the counter surface during the sliding test which led to increase in the distance between the base of the cantilever and the contact point. This may cause a variation in the applied load according to Equation (3). The degree of load variation was estimated by considering the stiffness and deflection of the cantilever. For the given suspension and the normal load of 20 mN, the typical deflection was 2 mm. Thus, based on the acceptable error of 10%, the variation of the depth due to wear of the specimen or the counter surface should not exceed 200 µm. This value was relatively large compared with the typical depth of wear encountered in the wear experiments.

### 2.6. Testing Conditions

To demonstrate the functionality of the in-situ tribotester developed in this work, friction and wear tests were performed. 10 × 10 mm^2^ silicon (Si) wafer coated with 50 nm thick SiO_2_ was used as the specimen and a stainless-steel ball with a diameter of 1 mm was used as the tip (counter surface). The ball was attached to the cantilever suspension using an epoxy adhesive. The test was performed at an ambient temperature of 24 °C and a relative humidity of ~45%. The normal force applied to the specimen was 20 mN, the length of the sliding stroke was 2 mm, and the reciprocating speed was 8 mm/s. The total number of sliding cycles was set to 500 and the reciprocating motion was paused every 50 cycles to measure and investigate the dynamics of the wear track generation without detaching the tip from the specimen surface.

## 3. Results

**Figure 7:**
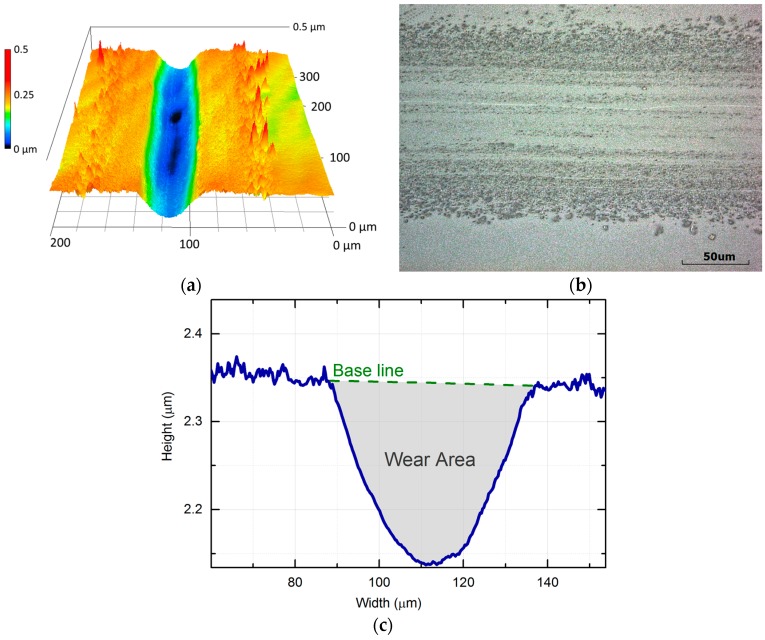
Example of the friction and wear experiment: (**a**) 3D topography image of the wear track; (**b**) corresponding optical image; and (**c**) cross-sectional profile of the wear track after 500 sliding cycles under a normal force of 20 mN.

The reciprocating sliding motion of the stainless-steel ball against the SiO_2_ coated Si specimen led to the formation of a distinct wear track. Figure 7a presents the 3D visualization of the wear track obtained by scanning the wear track using the laser microscope. Figure 7b shows a 2D cross-sectional profile of the wear track. The wear area could be calculated by integration of the region underneath the baseline that represented the original surface of the specimen.

**Figure 8:**
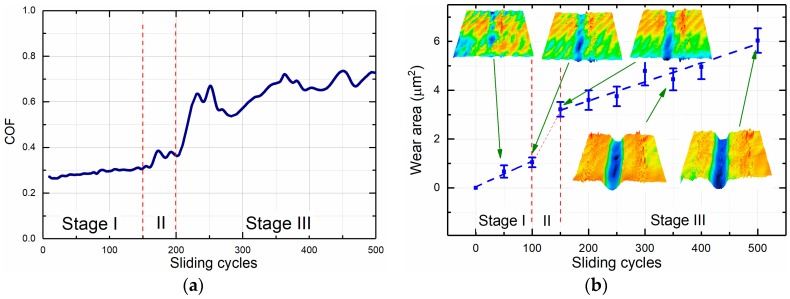
The result of the in-situ friction and wear experiment: (**a**) Example of the friction curve; and (**b**) wear area as a function of reciprocating sliding cycles. Insets: 3D topography images of the wear tracks at various intervals of the sliding test.

During sliding, the coefficient of friction between the tip and the specimen significantly varied as shown in Figure 8a. During the first ~160 cycles, it slowly increased from ~0.27 to ~0.33. After ~160 cycles the coefficient of friction increased rapidly up to ~0.6 and then continued to rise. Detailed analysis of the wear track topography, which was measured every 50 reciprocating cycles, significantly aided in explaining the non-monotonic frictional behavior. Figure 8b demonstrates the variation of the wear area as a function of sliding cycles. The 3D topography images of the wear track shown in the insets of Figure 8b correspond to different intervals of the friction and wear experiment. The dependence of the wear area on the number of sliding cycles was divided into three stages. During the first stage, the wear volume increased linearly up to 100 sliding cycles. During this stage, the wear track became wider and its depth increased. Nevertheless, after 100 cycles the depth of the wear track was lower than the thickness of the coating, which was 50 nm. Therefore, only the coating was worn out during this stage, and hence the wear rate was dictated by the wear behavior of the coating. The absence of large wear debris and smoothness of the wear track formed during this stage indicated that abrasive wear mechanism was not significant. Rather, it was presumed that adhesive wear was the main mechanism of wear in this stage.

The second stage occurred between 100 and 150 cycles and was characterized by a sharp increase in the wear volume. This significant increase in wear occurred because the tip penetrated through the coating and reached the interface between the coating and the Si substrate. The interface region was apparently much less wear resistant than both the coating and the substrate. This difference in the wear resistance could be attributed to the concentration of intrinsic residual stress at the SiO_2_/Si interface. It has been previously reported that internal residual stress exists at the SiO_2_/Si interface in SiO_2_ films grown by the thermal oxidation of silicon [24,25]. During the second stage, the formation of large wear debris was observed inside the wear track. Thus, transition of the wear mechanism from adhesion to abrasion occurred at this stage.

The third stage occurred after 150 cycles and was characterized by the wear volume continuing to increase linearly. The third stage corresponded to the penetration of the tip into the substrate. Formation of the deep wear scars was observed during this stage (Figure 7b). Thus, it was presumed that three-body abrasion dominated the wear process during this stage. As indicated in Figure 8b, the wear area variation with respect to sliding cycles for both stages I and III could be approximated by linear functions. The higher slopes in the first two stages indicate that the SiO_2_ coating and SiO_2_/Si interface have higher wear rates and hence lower wear resistance than the Si substrate. It was further postulated that three-body abrasion led to the higher coefficient of friction during stage III.

## 4. Conclusions

Wear is an important phenomenon that adversely affects the efficiency and lifespan of all machine components in contact under relative motion. In this regard, there is a need to develop new instruments to attain a better understanding of the wear phenomenon. In this paper, the design and experimental evaluation of a state-of-the-art in-situ tribotester were presented. The tribotester provided detailed real-time monitoring of the wear process by enabling quantitative analysis of the surface topography during various stages of the sliding process. Wear rates and wear track topography could then be correlated to the corresponding coefficients of friction, thereby enabling an in-depth elucidation of wear mechanisms at various stages. The effectiveness of the in-situ tribotester was demonstrated through friction and wear tests performed using a stainless-steel ball and a silicon wafer coated with SiO_2_. The transition of the wear mechanism during the sliding test were assessed.
